# Breed-dependent microRNA expression in the primary culture of skeletal muscle cells subjected to myogenic differentiation

**DOI:** 10.1186/s12864-018-4492-5

**Published:** 2018-01-31

**Authors:** Tomasz Sadkowski, Anna Ciecierska, Jolanta Oprządek, Edyta Balcerek

**Affiliations:** 1 Department of Physiological Sciences, Faculty of Veterinary Medicine, Warsaw University of Life Sciences – SGGW, Nowoursynowska 159, 02-776 Warsaw, Poland; 2 Department of Animal Improvement, Institute of Genetics and Animal Breeding of the Polish Academy of Sciences, Postępu 36A, Jastrzębiec, 05-552 Magdalenka, Poland

**Keywords:** microRNA, Myogenesis, Bovine satellite cells, Myoblast differentiation, Cattle

## Abstract

**Background:**

Skeletal muscle in livestock develops into meat, an important source of protein and other nutrients for human consumption. The muscle is largely composed of a fixed number of multinucleated myofibers determined during late gestation and remains constant postnatally. A population of postnatal muscle stem cells, called satellite cells, gives rise to myoblast cells that can fuse with the existing myofibers, thus increasing their size. This requires a delicate balance of transcription and growth factors and specific microRNA (miRNA) expressed by satellite cells and their supporting cells from the muscle stem cell niche. The role of transcription and growth factors in bovine myogenesis is well-characterized; however, very little is known about the miRNA activity during this process. We have hypothesized that the expression of miRNA can vary between primary cultures of skeletal muscle cells isolated from the semitendinosus muscles of different cattle breeds and subjected to myogenic differentiation.

**Results:**

After a 6-day myogenic differentiation of cells isolated from the muscles of the examined cattle breeds, we found statistically significant differences in the number of myotubes between Hereford (HER)/Limousine (LIM) beef breeds and the Holstein-Friesian (HF) dairy breed (*p* ≤ 0.001). The microarray analysis revealed differences in the expression of 23 miRNA among the aforementioned primary cultures. On the basis of a functional analysis, we assigned 9 miRNA as molecules responsible for differentiation progression (miR-1, -128a, -133a, -133b, -139, -206, -222, -486, and -503). The target gene prediction and functional analysis revealed 59 miRNA-related genes belonging to the muscle organ development process.

**Conclusion:**

The number of myotubes and the miRNA expression in the primary cultures of skeletal muscle cells derived from the semitendinosus muscles of the HER/LIM beef cattle breeds and the HF dairy breed vary when cells are subjected to myogenic differentiation. The net effect of the identified miRNA and their target gene action should be considered the result of the breed-dependent activity of satellite cells and muscle stem cell niche cells and their mutual interactions, which putatively can be engaged in the formation of a larger number of myotubes in beef cattle-related cells (HER/LIM) during in vitro myogenesis.

**Electronic supplementary material:**

The online version of this article (10.1186/s12864-018-4492-5) contains supplementary material, which is available to authorized users.

## Background

Skeletal muscle in livestock occupies approximately 40% of the animal body weight and develops into an important source of protein and other nutrients for human consumption [1, 2]. Skeletal muscle is largely composed of multinucleated muscle cells called myofibers. In cattle, the number of muscle fibers, a key determinant of the postnatal growth rate, is fixed late during the gestation period and remains constant postnatally, but each myofiber grows in size by the fusion of satellite cells [3, 4]. Activation of satellite cells (a population of postnatal muscle stem cells) give rise to myoblast cells that undergo multiple divisions before terminal differentiation. This can lead to fusion with the existing myofibers [5], which require a delicate balance between myogenic differentiation, myoblast proliferation, and the activity of transcription/growth factors and specific microRNA (miRNA). Signals from the surrounding cells, such as macrophages, fibroblasts, and muscle-resident stem cells support satellite cells in their action [6]. The effects of the involvement of transcription factors, such as MRF (MYF5, MYOD, myogenin, MRF4), and MEF2 families (reviewed in [7]), and the influence of the muscle stem cell niche on satellite cell behavior during skeletal muscle development is well-documented (reviewed in [8]), but little is known about the miRNA involvement in the regulation of this process, particularly in cattle.

Since the discovery of the first miRNA in 1993, the knowledge about miRNA’s impact on the development of different tissues has grown exponentially, including that in the skeletal muscle field [9, 10]. MicroRNA represents a class of ~ 22 nucleotide endogenous non-coding RNA molecules. These molecules modulate gene expression by base pairing to the 3’UTR regions of the target mRNA, leading to translational repression and/or mRNA cleavage [10]. Most of the miRNA regulating myogenic differentiation are found to be upregulated during this process [11]. Among them, the well-known muscle-specific miRNAs miR-1, -133a, -133b, and -206; also called myomiRs [11]. They play a fundamental role during muscle proliferation and differentiation by influencing a number of transcription factors and signaling molecules required for normal myogenesis progression.

Despite the increasing number of articles describing miRNA engagement in satellite cell activation, proliferation, and differentiation, the knowledge about their action, particularly in the development of bovine skeletal muscle, is still obscure. In this study, we focused on the differences in miRNA expression in a primary culture of skeletal muscle cells originating from the semitendinosus muscle of bulls of varying breeds and performance and subjected to myogenic differentiation.

## Methods

### Animals and tissue sampling

The experiment was conducted on primary cultures of skeletal muscle cells isolated from the semitendinosus muscle of 15-month-old bulls. The experimental groups were composed of Hereford (HER; high meat high-fat, maturating early, *n* = 4) and Limousin (LIM; high-meat low-fat, maturing late, *n* = 4) beef cattle bulls. Holstein-Friesian dairy cattle bulls (HF; typical dairy breed, maturing early with relatively poor carcass quality and a lower dressing percentage than beef bulls, *n* = 4) were used as the reference group. LIM and HER bulls were born in beef herds, while HF bulls in dairy herds. At the age of 2–3 months, the animals were transferred to the farm of Institute of Genetics and Animal Breeding Polish Academy of Sciences where they were housed in a loose barn until slaughter. The bulls were fed a total mixed ration (TMR) consisting of corn silage (75%), concentrates (20%), and hay (5%) and had access to water ad libitum [12, 13]. At the age of 15 months, all bulls were slaughtered after 24-h fasting. Samples of *m. semitendinosus* were immediately collected accordingly to the procedure described by Szcześniak et al. [14] and stored in liquid nitrogen until use.

### Skeletal muscle cell isolation, proliferation, and differentiation

The collected skeletal muscle tissue samples were thawed, washed in phosphate buffered saline (PBS; Sigma-Aldrich, USA) and suspended in the incubation medium: Dulbecco’s Modified Eagle Medium (DMEM; Life Technologies, USA), Pronase from *Streptomyces griseus* (Sigma-Aldrich, USA) at 0.5 mg/ml, and penicillinum crystallisatum TZF (Polfa Tarchomin, Poland) at 10000 IU/100 mL, and pH 7.3. The samples were incubated for 1.5 h in 37 °C, subjected to repeated pipetting every 15 min. Then, the suspension was filtered through a 70-μm cell strainer (Becton Dickinson, USA) to separate the tissue debris. The filtrate was centrifuged thrice (20 min, 350 *g*), and the pellet was resuspended each time in a growth medium (GM: 10% fetal bovine serum [FBS, Life Technologies, USA]/DMEM/1% penicillin-streptomycin and 0.5% amphotericin B). As muscle tissue is mainly composed of myofibers, we decided to use 1-h preplating to increase the number of myoblasts in the culture. The preplating method is based on the fact that myoblasts adhere to a polystyrene culture dish slower than other cells such as fibroblasts [15]. The first preplating was executed after the abovementioned final centrifugation. After 1-h of preplating, the supernatant was centrifuged and the pellet resuspended in GM, transferred into 25-cm^3^ Primaria tissue culture flasks (Becton Dickinson, USA), and cultured at 37 °C in 5% CO_2_ and 95% humidity. The growth medium was changed every 48 h. During cell proliferation, on day 6, 8, and 10, the cells were trypsinized, centrifuged, resuspended in GM, subjected to 1-h preplating, and sieved into the Primaria tissue culture flasks. The four-time preplating allowed for 60%–70% myoblast purity, measured as a percentage of the M-cadherin positive cells (data not shown) [16]. In brief, the cells were fixed with 4% parafomaldehyde (Sigma-Aldrich, USA) for 30 min and washed with PBS. They were incubated with monoclonal anti-M-cadherin (611,100, BD Biosciences, USA) at 1:100 dilution with PBS-1% BSA (Sigma-Aldrich, USA) for 1 h, and then, exposed to the AlexaFluor488-conjugated goat anti-mouse antibody (Invitrogen, USA) diluted 1:500 with PBS. Finally, the cells were incubated in a 7-AAD (Sigma-Aldrich, USA) solution for nuclei staining and observed with a FV-500 laser scanning confocal microscope (Olympus, USA). The number of M-cadherin positive cells was determined with respect to the total number of nuclei in the primary cell culture.

After the final passage, cells were transferred (100,000 per well) on to a 6-well Collagen I coated plate (Becton Dickinson, USA) and cultured in GM in 5% CO_2_ at 37 °C and 95% humidity. After reaching 80% cell confluence, GM was replaced by a differentiation medium (DM: 2%HS/DMEM/1% penicillin-streptomycin and 0.5% amphotericin B) in which cells were incubated for the next 6 days. DM was replaced every 48 h. On day 6, differentiated cells with visible myotubes were washed with PBS and stored at − 80 °C until the analysis.

### Myoblast fusion

To assess the number of myotubes on day 6 of the differentiation, the DM medium was discarded, cells were washed twice in ice-cold PBS, fixed in 75% methanol (15 min), stained with Giemsa dye (0.04% *w*/*v* in methanol; 5 min), and rinsed in distilled water (Sigma-Aldrich, USA). Nuclei were counted using a phase-contrast microscope (CK40, Olympus) randomly in five fields of view for each cell culture. Structures with at least three nuclei were considered myotubes (*n* = 4; see statistical analysis).

### RNA isolation and validation

Total RNA was extracted using the miRNeasy Mini Kit (Qiagen, Germany) according to the manufacturer’s protocol and validated using a Nanodrop spectrophotometer (Nanodrop Technologies, USA); its integrity was checked using a Bioanalyzer 2100 (Agilent Technologies, USA). Samples with RIN ≥ 9.5 were subjected to further analysis.

### miRNA microarray analysis

In the present study, custom Bovine miRNA microarrays (8 × 60 K) (Agilent Technologies, USA) containing probes for 763 *Bos taurus* miRNAs were used (National Center for Biotechnology Information Gene Expression Omnibus database (NCBI GEO): GPL19028). For the miRNA profiling, 100 ng of the total RNA from each sample (*n* = 4, see statistical analysis) was labeled and hybridized using miRNA Complete Labeling and Hyb Kit (Agilent Technologies, USA), according to the manufacturer’s protocol. RNA Spike-In Kit (Agilent Technologies, USA) was used as the internal control. Slides were scanned using Agilent Microarray Scanner (G2505C), and features were extracted using the Agilent Feature Extraction image analysis tool version 10.7.3.1 with default settings and default normalization scheme for Agilent one-color data (75th percentile scaling). The microarray data were statistically analyzed using Gene Spring 13 (Agilent Technologies, USA) and the default protocol for miRNA experiments, where one-way analysis of variance (ANOVA) with Benjamini-Hochberg multiple testing correction adjustment (FDR) was applied. MiRNAs with FDR ≤ 0.05 were selected as significantly differentially expressed. MiRNA with fold change of FC ≥ 2.0 were chosen as common for both HER/LIM primary cultures. The microarray experiment was performed according to the MIAME guidelines [17]. The data obtained in the microarray experiment were deposited in the NCBI GEO database and numbered GSE73778.

### qPCR validation

To validate the microarray results, selected miRNAs were examined using a real-time polymerase chain reaction technique (qPCR). First strand cDNA was synthesized using 10 ng of the total RNA (*n* = 4) and the miRCURY LNA™ Universal RT cDNA Synthesis Kit II (Exiqon, Denmark) with UniSp6 Spike-in used as the internal control. qPCR analyses were performed using a SYBR® Green master mix, Universal RT (Exiqon, Denmark), as follows: polymerase activation at 95 °C for 10 min; amplification (40 cycles) including denaturation at 95 °C for 10 s, annealing at 60 °C for 1 min; melting curve included: denaturation at 95 °C for 0 s, annealing at 65 °C for 15 s, continuous melting at 95 °C for 0 s; and cooling at 40 °C for 30 s. Primers for the selected miRNA and reference U6 snRNA were provided by Exiqon (Denmark). Their sequences and miRBase accession numbers are listed in Table 1. Each sample was tested twice with a Stratagene Mx3005P thermal cycler (Agilent Technologies, USA).

**Table 1:** miRNA primers used for validation of microarray results (Exiqon, Denmark)

miRNA Primer	Target sequence	Accession (Exiqon)
*hsa-miR-1 LNA™ PCR primer set, UniRT*	UGGAAUGUAAAGAAGUAUGUAU	MIMAT0000416
*bta-miR-9-5p LNA™ PCR primer set, UniRT*	UCUUUGGUUAUCUAGCUGUAUG	MIMAT0009389
*hsa-miR-128-3p LNA™ PCR primer set, UniRT*	UCACAGUGAACCGGUCUCUUU	MIMAT0000424
*hsa-miR-133a-3p LNA™ PCR primer set, UniRT*	UUUGGUCCCCUUCAACCAGCUG	MIMAT0000427
*hsa-miR-139-5p LNA™ PCR primer set, UniRT*	UCUACAGUGCACGUGUCUCCAGU	MIMAT0000250
*hsa-miR-145-5p LNA™ PCR primer set, UniRT*	GUCCAGUUUUCCCAGGAAUCCCU	MIMAT0000437
*hsa-miR-206 LNA™ PCR primer set, UniRT*	UGGAAUGUAAGGAAGUGUGUGG	MIMAT0000462
*hsa-miR-486-5p LNA™ PCR primer set, UniRT*	UCCUGUACUGAGCUGCCCCGAG	MIMAT0002177
*cfa-miR-503 LNA™ PCR primer set, UniRT*	UAGCAGCGGGAACAGUACUG	MIMAT0006746
*bta-miR-660 LNA™ PCR primer set, UniRT*	UACCCAUUGCAUAUCGGAGCUG	MIMAT0004344
*U6 snRNA LNA™ PCR primer set, UniRT* *(reference RNA)*	GUGCUCGCUUCGGCAGCACAUAU ACUAAAAUUGGAACGAUACAGAG AAGAUUAGCAUGGCCCCUGCGCA AGGAUGACACGCAAAUUCGUGAA GCGUUCCAUAUUUUU	without MIMAT #

A qPCR analysis for selected myogenesis-related genes that could interact with the identified miRNAs, such as myogenin (*Myog*, accession no. NM_001111325), *Myod* (accession no. NM_001111325), *Myf5* (accession no. NM_001111325), and *Mstn* (accession no. NM_001111325), was also performed. For this purpose, primers were designed using the Primer3 software and *Bos taurus* mRNA sequences obtained from the NCBI Nucleotide database. Specificity of primers and secondary structure formation were checked as described previously [18]. First strand cDNA synthesis was performed using 1 μg of the total RNA and the Transcriptor First Strand cDNA Synthesis Kit (Roche, USA) with a mixture of oligo(dT)18 and random hexamer primers, according to the manufacturer’s protocol. qPCR primers sequences and conditions are listed in Additional file 1. The analysis was performed according to the methodology described earlier with glyceraldehyde-3-phosphate dehydrogenase (*Gapdh*, accession no. NM_001034034) used for normalization as the non-regulated reference gene [19]. Each sample was tested twice in a Stratagene Mx3005P thermal cycler (Agilent Technologies, USA). The amplification efficiency (E = 10(− 1/slope) - 1) was determined by plotting a comparative quantitation standard curve and was ≥0.9 for each gene. The qPCR analysis was conducted according to a standardized approach [20].

### Target gene prediction

Target genes for differentially expressed miRNA were identified using TargetScan release 7.1 (http://www.targetscan.org/vert_71/). The TargetScan database was chosen because of the availability of bovine species for prediction. Scanning was performed for the target score (total context score) of ≤ − 0.3 and conserved/non-conserved miRNA families and target sites. Moreover, the Pathway Studio Web (Elsevier, Netherlands) with Enrichment Analysis of Selected Entities tool was also used for target gene searching. In the Pathway Studio Web analysis, the literature data were considered, proving real miRNA targets, or if not available, prediction was done on the basis of the PicTar and miRanda databases. Only genes found in both analyses were considered targets for the identified miRNAs.

### Functional analysis

An ontological analysis of miRNAs and their direct/indirect target genes was done using The Database for Annotation, Visualization and Integrated Discovery (DAVID) v6.7 tool (http://david.ncifcrf.gov/) where Gene Ontology Enrichment was calculated using the EASE Score corrected for multiple hypotheses testing using the Benjamini and Hochberg false discovery rate (FDR ≤ 0.05). Further, a similar analysis was performed using the PANTHER database (http://pantherdb.org/) and the functional classification tool. The relevance networks of the identified miRNAs and the selected target genes were prepared in Pathway Studio Web by using the Pathway Builder tool.

### Statistical analysis

A statistical analysis of the differences in the number of myotubes was performed using one-way ANOVA with Tukey post-hoc testing and a *p* value of ≤0.05. The microarray data analysis was performed using Gene Spring 13 (Agilent Technologies, USA) as described above. The miRNA and mRNA qPCR results were analyzed by the GenEx 6.0 (MultiD Analyses AB, Sweden) and Prism 5.0 (GraphPad Software, USA) software using the comparative Ct method [21]. Results with *p* ≤ 0.05 were considered to be statistically significant. Hierarchical clustering (Ward’s algorithm with Euclidean distance measure) and Spearman’s correlation coefficients were calculated using GenEx 6.0 (MultiD Analyses AB, Sweden). For each animal in the experimental groups (*n* = 4), two independent cell isolations were made (one from each half-carcass). Results from both half-carcasses were averaged for the animal, and then, the interbreed comparison and the statistical analysis were performed. In case of Gene Spring analysis, because of software limitations, it was not possible to average microarray results from both half-carcasses (two separate arrays) before the data processing. Gene Spring analysis was performed using 8 samples for each breed (4 animals × 2 half-carcasses).

## Results

### Differences in myotube formation in vitro

**Fig. 1:**
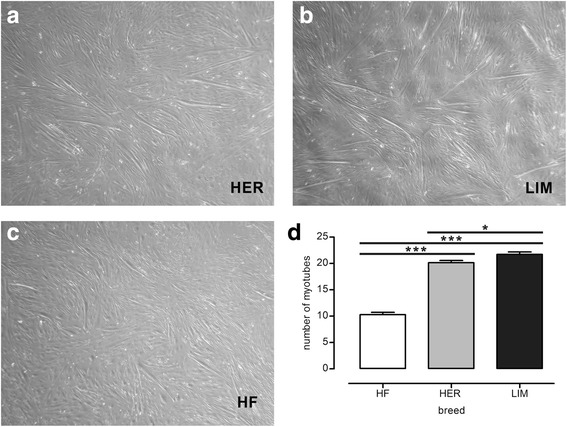
Myotube formation on day 6 of the differentiation of the primary cultures of skeletal muscle cells originating from the semitendinosus muscle of HER (**a**), LIM (**b**), and HF (**c**) breeds. Bar graph (**d**) presenting the difference in the number of myotubes between the examined cell cultures; results are shown as mean ± SEM with * and *** asterisks for *p* ≤ 0.05 and *p* ≤ 0.001, respectively; *n* = 4

The myotube formation assessment on day 6 of the skeletal muscle cell differentiation revealed statistically significant differences in the number of myotubes among all examined breeds. The difference between cells originating from HER/LIM and HF bulls’ muscle was statistically significant (*p* ≤ 0.001) with a considerable increase in the number of myotubes in beef cattle-related cell cultures. A difference between both beef breeds was also noticed (*p* ≤ 0.05) (Fig. 1).

### Microarray analysis

**Fig. 2:**
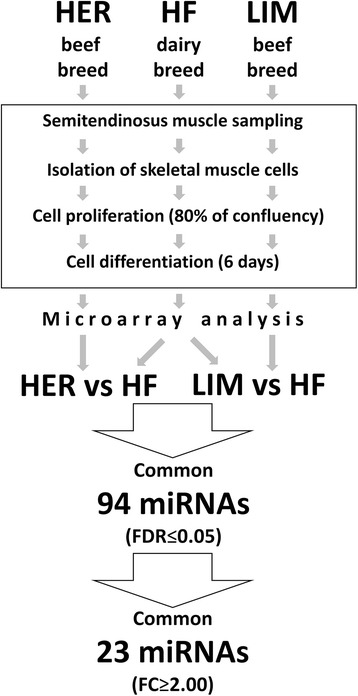
Experiment design and microarray results. HF - Holstein-Friesian; HER - Hereford; LIM - Limousine; *n* = 4

The microarray analysis of the total RNA isolated from the aforementioned cell cultures revealed differences in the expression of 94 miRNAs (FDR ≤ 0.05) in the primary cultures originating from both beef breeds HER/LIM, when compared with the HF dairy breed-derived cells (Additional file 2). The 23 molecules whose expression was changed at least twice in both comparisons (HER vs. HF and LIM vs. HF; FDR ≤ 0.05, FC ≥ 2.0; both up- or downregulated) were classified as the miRNAs characteristic for beef cattle-originating primary cultures of skeletal muscle cells and as candidate molecules responsible for the significant increase in the number of myotubes observed in the HER/LIM cultures (Fig. 2). Among them, 17 miRNA molecules had an increased level of expression and 6 had a lower miRNA expression in the HER/LIM cells. The microarray results showed that the most upregulated expression changes were noticed for miR-139, miR-2469, and miR-486, while the highest downregulation was observed for miR-9, miR-29b, and miR-31 (Table 2). Further, as the number of myotubes varied between both beef breeds, a statistical analysis of the microarray results was performed for this comparison as well and revealed no differences in the miRNA expression between the HER and the LIM-derived cell cultures (FDR ≤ 0.05, *t*-test).

**Table 2:** MiRNAs varying in expression in primary cultures of skeletal muscle cells isolated from semitendinosus muscle of both beef breed bulls (HER and LIM), when compared with the dairy breed (HF; the reference)

No.	Systematic name	p (Corr)	FC (HER vs. HF)	Log FC (HER vs. HF)	Regulation (HER&LIM vs. HF)	FC (LIM vs. HF)	Log FC (LIM vs. HF)	Active Sequence	miRBase Accession No.
1	bta-miR-139	3.07E-13	123.08	6.94	up	126.11	6.98	ACTGGAGACACGTGC	MIMAT0003788
2	bta-miR-2469	1.07E-03	83.81	6.39	up	11.25	3.49	AAGCCGCAGGCCC	MIMAT0012059
3	bta-miR-486	2.06E-03	51.74	5.69	up	35.07	5.13	CTCGGGGCAGCTCA	MIMAT0009329
4	bta-miR-2439-3p	6.58E-03	31.43	4.97	up	7.98	3.00	TCTGCCTACCTGTCTTC	MIMAT0012014
5	bta-miR-449a	4.82E-02	21.21	4.41	up	10.53	3.40	ACCAGCTAACAATACACTGC	MIMAT0009320
6	bta-miR-503-5p	1.26E-02	15.69	3.97	up	15.71	3.97	CAGTACTGTTCCCGC	MIMAT0025557
7	bta-miR-1	1.87E-06	12.44	3.64	up	14.67	3.87	ATACATACTTCTTTACATTCC	MIMAT0009214
8	bta-miR-133a	1.68E-06	11.63	3.54	up	12.57	3.65	CAGCTGGTTGAAGGGGAC	MIMAT0009225
9	bta-miR-206	1.07E-05	11.60	3.54	up	13.36	3.74	CCACACACTTCCTTAC	MIMAT0009260
10	bta-miR-133b	5.70E-06	11.53	3.53	up	12.12	3.60	TAGCTGGTTGAAGGGGACC	MIMAT0009226
11	bta-miR-542-5p	2.59E-02	8.77	3.13	up	25.04	4.65	CTCGTGACATGATGATC	MIMAT0013594
12	bta-miR-128	2.59E-02	4.51	2.17	up	4.78	2.26	AAAGAGACCGGTTCACTGT	MIMAT0003541
13	bta-miR-660	5.18E-04	2.49	1.32	up	2.26	1.18	CAGCTCCGATATGCAA	MIMAT0004344
14	bta-miR-378b	2.00E-03	2.42	1.28	up	2.38	1.25	GCCTTCTGACTCCAAG	MIMAT0025535
15	bta-miR-378c	4.33E-03	2.25	1.17	up	2.18	1.13	ACTTCTGACTCCAAGTC	MIMAT0025551
16	bta-miR-30a-5p	6.14E-03	2.14	1.10	up	2.51	1.33	AGCTTCCAGTCGAGG	MIMAT0003841
17	bta-miR-30f	1.45E-02	2.14	1.10	up	2.86	1.52	AGCTGAGAGTGTAGGGT	MIMAT0009282
18	bta-miR-9	2.77E-02	−40.65	−5.35	down	−16.45	−4.04	TCATACAGCTAGATAACCA	MIMAT0009389
19	bta-miR-29b	2.08E-03	−18.13	−4.18	down	−4.24	−2.08	AAACACTGATTTCAAATGGT	MIMAT0003828
20	bta-miR-31	1.99E-03	−16.57	−4.05	down	−213.81	−7.74	AGCTATGCCAGCATCTT	MIMAT0003548
21	bta-miR-194	4.25E-02	−14.83	−3.89	down	−2.55	−1.35	TCCACATGGAGTTGCT	MIMAT0009254
22	bta-miR-145	3.22E-02	−3.38	−1.76	down	−2.82	−1.5	AGGGATTCCTGGGAAAAC	MIMAT0003542
23	bta-miR-222	4.74E-02	−2.53	−1.34	down	−2.12	−1.08	ACCCAGTAGCCAG	MIMAT0003530

*FC* fold change, *HF* Holstein-Friesian, *HER* Hereford, *LIM* Limousine; FDR ≤ 0.05; FC ≥ 2.0; *n* = 4 (see statistical analysis)

### Functional analysis of identified miRNAs

**Fig. 3:**
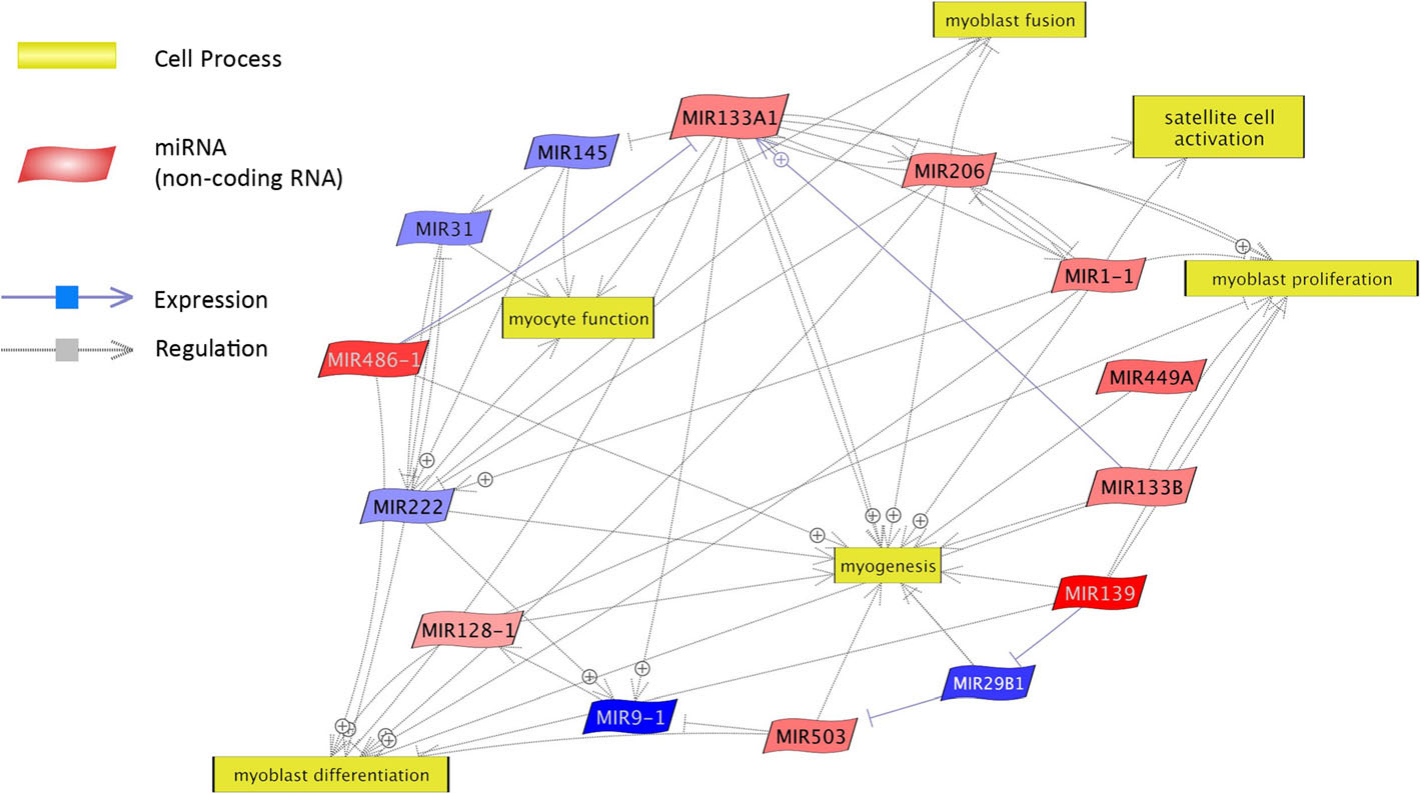
Relevance network of identified miRNAs and biological processes concerning skeletal muscle development. Upregulated and downregulated miRNAs were marked in red and blue, respectively (Pathway Studio Web; Elsevier, Netherlands)

A functional analysis of 23 identified miRNA molecules using Pathway Studio Web (Elsevier, Netherlands) allowed the identification of muscle development-related biological processes in which HER/LIM cell-specific miRNAs could be involved. According to the aforementioned database, a majority of the analyzed molecules were engaged in the myogenesis process (miR-1, -29b, -128, -133a, -133b, -139, -206, -222, -449a, -486, and -503). Among them, 9 miRNA were classified as involved in myoblast differentiation (miR-1, -128, -133a, -133b, -139, -206, -222, -486, and -503). Moreover, some of identified molecules were also annotated as taking part in myoblast proliferation (miR-1, -128, -133a, -133b, -139, and -206); myocyte function (miR-31, -133a, -145, and -222); myoblast fusion (miR-206, -222, and -486); and satellite cell activation (miR-1 and -206) (Fig. 3).

### Real-time qPCR validation

**Fig. 4:**
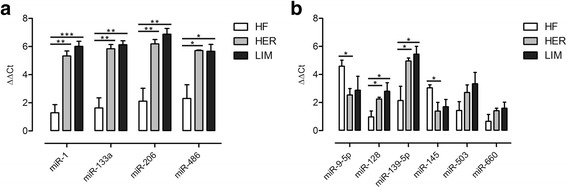
Real-time qPCR validation of microarray results for myomiRs (**a**) and non-myomiRs (**b**). Results are shown as mean ± SEM with *, **, and *** asterisks for *p* ≤ 0.05, *p* ≤ 0.01, and *p* ≤ 0.001, respectively; *n* = 4

Based on the available literature and the Pathway Studio Web analysis, 10 out of the 23 miRNAs identified by the microarray technique were selected for the qPCR validation. Among them were muscle-specific myomiRs miR-1, -133a, -206, and miR-486 and non-myomiRs such as miR-9-5p, -128, -139, -145, -503, and -660. A statistical analysis of the qPCR results using the GenEx 6.0 software confirmed microarray data showing a significant upregulation of myomiRs in the HER/LIM cell cultures as compared to that in the HF cell cultures (Fig. 4A). For non-myomiRs, a statistically significant upregulation of miR-128 and -139 was confirmed. Statistically significant downregulation was confirmed for miR-9-5p and miR-145 only in the case of the HER cell cultures (for LIM, *p* ≤ 0.16 and *p* ≤ 0.053, respectively). The expression difference of miR-503 and miR-660 was not statistically significant in the qPCR analysis (Fig. 4B). All miRNAs validated by qPCR exhibited the same trend of expression change as in the microarray experiment.

### Expression of selected myogenesis-related genes

**Fig. 5:**
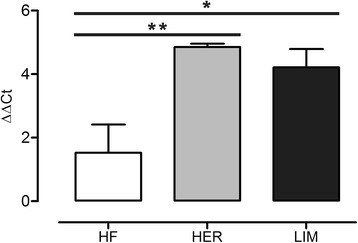
Expression of myogenin mRNA in primary cultures of skeletal muscle cells at 6th day of differentiation. Results are shown as mean ± SEM with * and ** asterisks for *p* ≤ 0.05 and *p* ≤ 0.01, respectively; *n* = 4

Based on previous publications, a few pivotal myogenesis-related genes (*Myod*, *Myf5*, myogenin and *Mstn*) described as influenced by some of the identified miRNAs were chosen. The qPCR analysis revealed an increased transcript level in both HER/LIM-derived cells only for myogenin mRNA (Fig. 5). The difference in the myogenin expression between HER and LIM was not statistically significant. Expression of the other examined genes was not statistically significant in both HER/LIM cell cultures as compared to that of the HF-derived cells.

### Hierarchical clustering and Spearman’s correlation coefficient

**Fig. 6:**
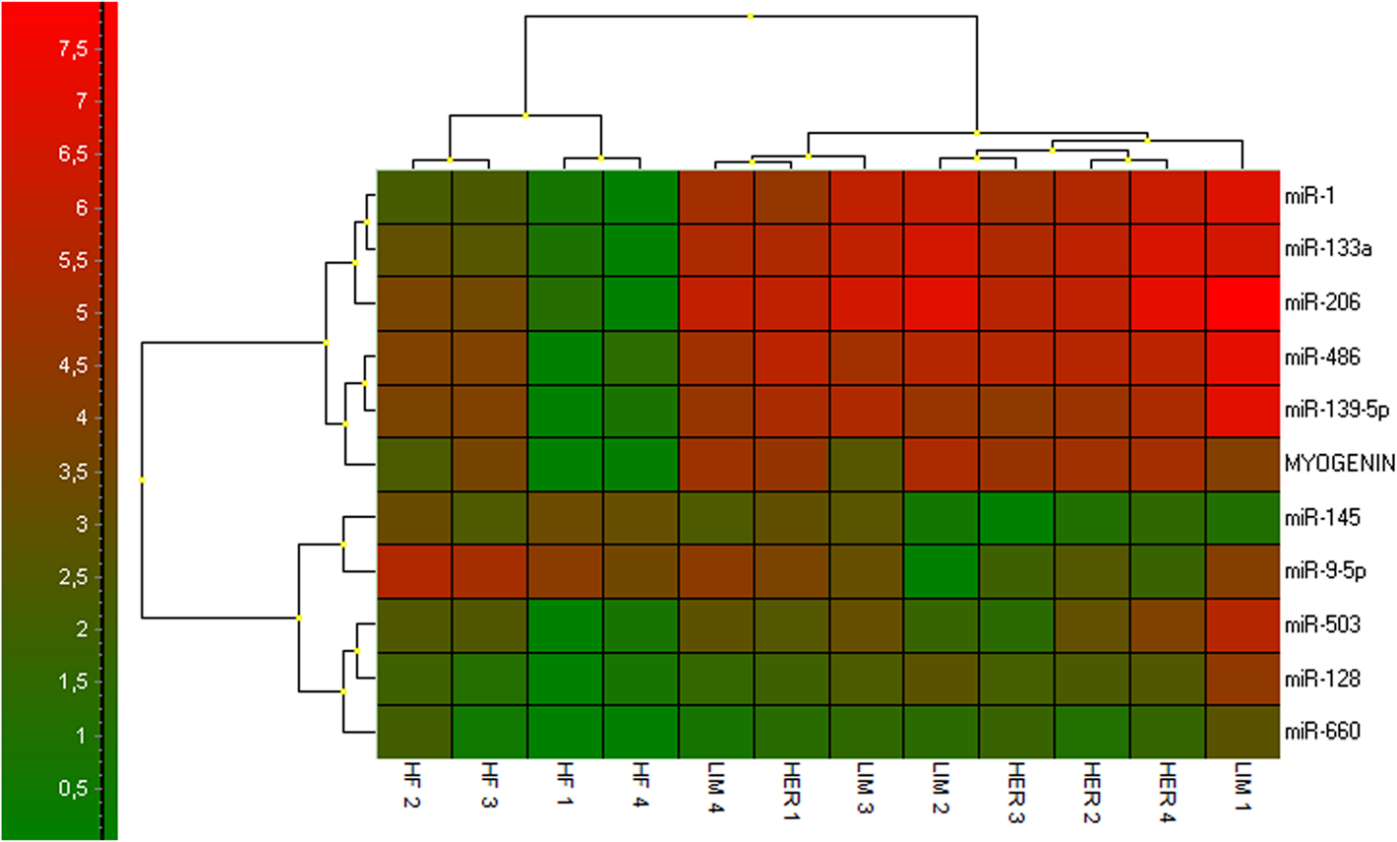
Hierarchical clustering of the validated miRNAs and myogenin (GenEx 6.0; MultiD Analyses AB, Sweden); *n* = 4

Hierarchical clustering for the validated miRNAs and myogenin was performed using GenEx 6.0 (MultiD Analyses AB, Sweden). A clear distinction was observed between the beef and the dairy cattle-derived cell cultures, showing a higher similarity between HER and LIM, than HF cells (Fig. 6). Further, Spearman’s correlation coefficients calculated for the validated miRNAs showed a strong or very strong uphill correlation among the myomiRs (miR-1, -133a, -206, and -486), miR-128, and miR-139. All the aforementioned correlations were significant with *p* ≤ 0.01 (Additional file 3). Moreover, strong uphill correlations were also shown for the aforementioned myomiRs and myogenin with *p* ≤ 0.05 (Additional file 3).

### miRNA target gene prediction and functional analysis

The TargetScan database and the Pathway Studio algorithm (PicTar and miRanda) were used for the miRNA target gene prediction. TargetScan search resulted in 5142 records (total context score ≤ − 0.3) with 3918 unique targets (after the removal of duplicates). The Pathway Studio analysis revealed 3355 records and 2392 unique target genes for the set of the 23 identified miRNA molecules. Both sets of genes were compared, and the overlapping 1249 miRNA targets were considered significant (Additional file 4). The batch of all identified targets was loaded in the DAVID online software to find biological processes in which miRNA-related genes are involved. The functional analysis resulted in 192 processes (FDR ≤ 0.05) in which nearly 1250 genes were engaged (Additional file 5). Among them, 40 genes were involved in the muscle organ development process (Table 3). Moreover, the PANTHER online database was used for a functional analysis for the same group of targets, showing the involvement of 21 genes in the muscle organ development process (Table 3, Additional file 6). Both sets of targets belonging to the developmental process of a muscle organ were combined into one group of miRNA-related genes of interest. Surprisingly, only 5 target genes were similar for both analyses (DAVID/PANTHER) (Table 3).

**Table 3:** Muscle organ development process-related target genes identified in DAVID and PANTHER. Full sets of identified processes and the corresponding target genes are listed in Additional files 5 (DAVID) and 6 (PANTHER)

Selected muscle-related biological processes (DAVID)			
Term	Count	Genes	FDR
GO:0007517 muscle organ development	40	*Sri, Cav2,* *Mef2a* *, Utrn, Eln,* *Cacnb2* *,* *Pax3* *, Tagln2, Itgb1, Pten, Tgfb2, Gphn, Atg5, Gata6, Hlx,* *Pax7* *, Col6a3, Rhoa, Ppp3ca, Rarb, Nr2f2, Col11a1, Foxl2, Actc1, Met, Mstn, Igf1, Tbx1,* *Mbnl1* *, Sirt1, Prox1, Foxp1, Foxp2, Fxr1, Mapk14, Six1, Pdgfrb, Hbegf, Foxc1, Serp1*	1.11E-04
Selected muscle-related biological process (PANTHER)			
Term	Count	Genes	
muscle organ development (GO:0007517)	21	*Hoxa9, Lef1, Otx2, Lpp,* *Pax7* *, Hoxb4, Lhx5, Hoxa11, Dlx3, Myo1c, Pdlim2, Myo5a, Myo10,* *Cacnb2* *,* *Mef2a* *, Hoxa13, Pax3, Lhx8, Cdh24, Hoxc13,* *Mbnl1*	

Underlined are genes common for both the DAVID and the PANTHER analyses. *DAVID* The Database for Annotation, Visualization and Integrated Discovery v6.7, *PANTHER* Protein ANalysis THrough Evolutionary Relationships Classification System, *FDR* false discovery rate

Apart from their involvement in the muscle organ development process, the identified target genes were classified as participating in positive (49) and negative (45) regulation of cell differentiation. Target genes belonging to all the identified processes are listed in Additional file 5.

Using the DAVID algorithm, the Kyoto Encyclopedia of Genes and Genomes (KEGG) database was searched, showing the involvement of the predicted targets in 56 signaling pathways (Additional file 7). This included MAPK, insulin signaling, mTOR, ErbB, TGFβ, and Wnt pathways (Additional file 8), which are mentioned in discussion as possibly regulated by the identified miRNAs.

## Discussion

MiRNAs and their role in skeletal muscle development have been studied in recent years showing that myogenesis is governed not only by myogenic regulatory factors, (such as MYF5, MYOD, myogenin, MRF4), growth factors and the related proteins, but also by small non-coding RNA. These small non-coding RNA fragments cooperate with the abovementioned regulatory factors in executing myogenic cell proliferation, differentiation, fusion, and myofiber maturation during skeletal muscle growth [8]. Albrecht et al. [22, 23] in their in vivo study reported a tendency showing a higher number of muscle fibers per muscle bundle in beef than in dairy cattle during both late gestation and postnatal muscle maturation. However, a statistically significant difference was observed only in the case of the Belgian Blue breed when compared to Holstein-Friesian. Rehfeldt et al. [24] suggested that animals with a larger number of muscle fibers of moderate size produce more meat of better quality. In a previous publication, we reported the major carcass traits of three cattle breeds, with the lowest dressing percentage for the Holstein-Friesian breed and significantly higher for the Hereford and Limousin breeds [12].

Because muscle mass is determined, at least in part, by the number of muscle fibers already formed during fetal life [4], we decided to examine the differences in the in vitro differentiation of skeletal muscle cells between beef and dairy cattle-derived primary cultures and to investigate the breed-specific miRNA pattern characteristic for HER/LIM cell cultures.

In the proposed experimental model, we did not separate all the non-myogenic cells from the culture to allow the influence of the muscle stem cell niche cells on the satellite cells present in the primary culture. As the cells were isolated from the same portion of the semitendinosus muscle, in two independent isolations from both half-carcasses of each animal, using the same amount of tissue and the same methodology, we considered such prepared primary cultures as characteristic for the specific breed. To increase the number of satellite cells that we wanted to subject to differentiation in the primary cultures, the preplating procedure was used four times. The preplating was done in the same manner for all the isolations from all the animals. This approach should provide a condition similar to this in muscle in vivo (except the number of myoblast cells), where proliferating, differentiating, and fusing myoblasts are under the influence of the factors and metabolites released by neighboring cells representing the skeletal muscle stem cell niche [8]. It must be kept in mind that the considered primary cultures of skeletal muscle cells were not pure satellite cell cultures but contained an enhanced percentage of myogenic cells and different breed-dependent amount of other cells present in the muscle tissue. This could be a main source of variation of the myotube number and miRNA expression in the examined primary cultures of skeletal muscle cells.

As a result of the myogenic differentiation of the isolated cells, enhanced myotube formation was observed in the HER/LIM-derived primary cultures as compared to the HF cells (Fig. 1). It was accompanied by a differential expression of the 23 miRNAs (FDR ≤ 0.05, FC ≥ 2.0; Fig. 2, Table 2). Real-time qPCR validation of the selected miRNA confirmed the trends observed in the microarray analysis; however, the expression differences of miR-503 and miR-660 were not statistically significant (Fig. 4). Moreover, a higher expression of myogenin mRNA was detected in both HER/LIM breed-related cells (Fig. 5). A functional analysis of the identified miRNAs confirmed their involvement in myogenesis, particularly in the differentiation processes (Fig. 3). Considering the above data, in the following discussion, it should be kept in mind that the obtained results relate to the interbreed differences in the miRNA expression in the primary cell cultures subjected to differentiation, and they are not a proliferation vs. differentiation comparison. It is also important to note that the isolated cells were not pure satellite cell cultures and the interbreed differences in myogenic/non-myogenic cell content in the primary culture could be the main source of the observed variation in the miRNA expression.

In the following discussion, symbols of genes and their protein products have been presented in their original form depending on the specificity of the cited paper, as lowercase italic with the first letters in uppercase or as uppercase letters when they refer to transcriptomic or proteomic research, respectively.

A few high-throughput studies have confirmed some of the identified miRNAs (miR-1, miR-128, miR-133a, miR-133b, miR-206, miR-222, and miR-503) as common for skeletal muscle development in mouse, human, pig, common carp [11], and cattle [25]. All the aforementioned miRNAs have manifested upregulation during myogenic differentiation, except the downregulated miR-222, both in the above-cited papers [11, 25] and in our study (Table 2).

### MyomiRs - muscle-specific miRNAs

In our experiment, four myomiRs were identified as differentially expressed. Their expression in the HER/LIM-derived primary cultures was considerably higher than in HF cells (Table 2, Fig. 4). In general, the myomiRs expression is greatly enhanced during myogenesis and is required for its optimal progression [26]. Cell culture experiments have shown that miR-1 and miR-206 promote muscle cell differentiation, whereas miR-133 enhances cell proliferation. Dai et al. [27] confirmed the mechanism in which miR-1 and miR-206 positively regulate bovine skeletal muscle satellite cell myogenic differentiation via the downregulation of PAX7 and HDAC4. MiR-1 and miR-206 were also found to inhibit PAX3 [28] and NOTCH3 [29] allowing differentiation to proceed. Moreover, miR-206 directly targets cyclin D1 (CCND1) and DNA polymerase α (POLA1), reducing the proliferation rate of myogenic cells [30]. It might also indirectly downregulate DNA-binding protein inhibitors (IDs) and myogenic repressors (MYORs), the *Myod* inhibitors, and thus, regulate myoblast differentiation [10]. One study suggested that the rapid removal of SNAI1 and SNAI2 at the onset of differentiation is mediated by miR-30a and miR-206, respectively, resulting in the upregulation of myogenin and a dependent increase in the miR-30a and miR-206 expression [31]. Confirmed upregulation of myogenin mRNA in the HER/LIM cells could strengthen the possibility that similar mechanisms are executed in these cells (Fig. 5). It should be noted that miR-30a, which regulates the *Snai1* expression, was also upregulated in our study (Table 2).

MiR-133, another myomiR, has been confirmed to increase myoblast proliferation and regulate differentiation by targeting SRF, MAML1, nPTB, and UCP2 proteins [10, 32]. The combined action of miR-133 and myomiRs (miR-1 and -206) induces MYOD1, PAX7, and myogenin causing myoblast differentiation [33]. Upregulation of both miR-133 and myogenin under IGF-1 influence has also been described [34]. It is plausible that in HER/LIM cells, the differentiation progression is accelerated via similar mechanisms involving miR-1, miR-133, miR-206, and myogenin, resulting possibly in enhanced myotube formation observed in the primary cultures of the skeletal muscle with a HER/LIM origin (Fig. 1).

Finally, miR-486 was last of the myomiRs differing in expression between the HER/LIM and the HF cells (Table 2, Fig. 4). MiR-486 has been shown to influence myoblast differentiation by targeting *Pax7* and promoting the IGF-1R/PI3K/AKT signaling pathway by repressing its negative regulators PTEN and FOXO1A [28, 35]. PTEN inhibition leads to the activation of mTOR and an increase in protein synthesis [36]. The downregulation of miR-486 in normal myoblasts results in an impaired migration and myoblast fusion [37].

Note that the feedback loop of transcription factors, including myogenin, has been confirmed to participate in myomiR regulation [38], which may indicate it to be a molecule specifically modulating the miRNA expression in HER/LIM-derived cells, which can in turn be reinforced by strong uphill correlations between myogenin and the myomiRs identified in this study (Fig. 6, Additional file 3).

### Non-myomiRs engaged in myoblast differentiation

In addition to the aforementioned myomiRs, some of the identified miRNAs are also linked to myogenic differentiation. Among them is miR-139, which was expressed over 120 times more in the HER/LIM cells (Table 2, Fig. 4). Hasseine et al. [39] found that miR-139 directly targets *Foxo1* mRNA and reduces the level of its protein. It is plausible that miR-139 supports the miR-486-dependent inhibition of *Foxo1* translation, thereby increasing the mTOR-mediated protein synthesis. However, information about its role in skeletal muscle development is still scarce. Moreover, miR-139-5p was also shown to induce cell cycle arrest (prerequisite of differentiation) by targeting oncogenic nuclear receptor subfamily 5, group a, member 2 (*Nr5a2*) [40], which was confirmed to be developmentally regulated in bovine skeletal muscle [41].

Another molecule, miR-128a (Table 2, Fig. 4), known to be increased during myoblast differentiation, was upregulated in cells derived from beef cattle muscle. Overexpression of miR-128a in myoblasts impede cell proliferation by targeting IRS1 [42], while its inhibition promotes proliferation and myotube hypertrophy [43]. It has also been found to regulate the target genes involved in insulin signaling. MiR-128a is predicted to target the 3′-untranslated region of *Foxo1* [43] and thus, regulate AKT signaling, which could potentiate the action of the earlier described miRNAs -139 and -486. Another miR-128 putative target engaged in muscle development, *Nr5a2*, was identified in chickens [44]. Further, we assume that, at least partially, the coordinated action of miR-128 and miR-139 could influence the HER/LIM cell differentiation in a similar manner, possibly via the inhibition of *Foxo1* and *Nr5a2*. Both the aforementioned miRNAs have manifested strong and very strong uphill correlations between each other and myomiRs, respectively, and a moderate uphill correlation with myogenin (Fig. 6, Additional file 3).

The last miRNA in this group is miR-222. It has been downregulated in HER/LIM primary cultures. Cardinali et al. [45] reported miR-222 decrease in the differentiated quail myotubes. They confirmed the correlation of the miR-222 expression with the Ras/MAPK pathway and showed that its inhibition induced the expression of p27 (cell cycle inhibitor) and muscle-specific proteins, facilitating cell fusion and the assembly of contractile structures [45]. In recent studies, they also found that the overexpression of miR-222 and the consequent silencing of *Rbm24* resulted in the inhibition of myoblast fusion [46]. Furthermore, it was found that miR-222 negatively contributes to myoblast differentiation, taking part in the regulatory axis that includes mTOR and IGF-II [47]. miR-221/222 overexpression could directly increase β-catenin and repress the Wnt pathway inhibitors, enhancing the activity of the classic Wnt/β-catenin signaling pathway, which was shown to induce satellite cell proliferation [48].

It seems that all the aforementioned miRNAs differentially expressed in the HER/LIM-originating primary cultures of skeletal muscle cells can direct myogenesis towards differentiation progression (by different but complementary mechanisms), which can result in the increased number of myotubes observed in the HER/LIM-related cell cultures (Fig. 1).

### Other miRNAs

Besides the abovementioned miRNAs promoting myoblast differentiation, a few identified molecules such as miR-29b [49], miR-31 [50], miR-9 [51], miR-145 [52], miR-194 [53], miR-378 [54], miR-449 [55], miR-503 [11, 27], miR-542, [56], and miR-660 [11] were described in the literature as skeletal muscle-related. At this time, sufficient data have not been published concerning miR-2439-3p and miR-2469 engagement in the proliferation and differentiation processes of any cells. Their increased expression, particularly in HER-related cell cultures, suggests that they could play an important role in in vitro myogenesis in this breed.

### Myogenesis-related miRNA target genes

**Fig. 7:**
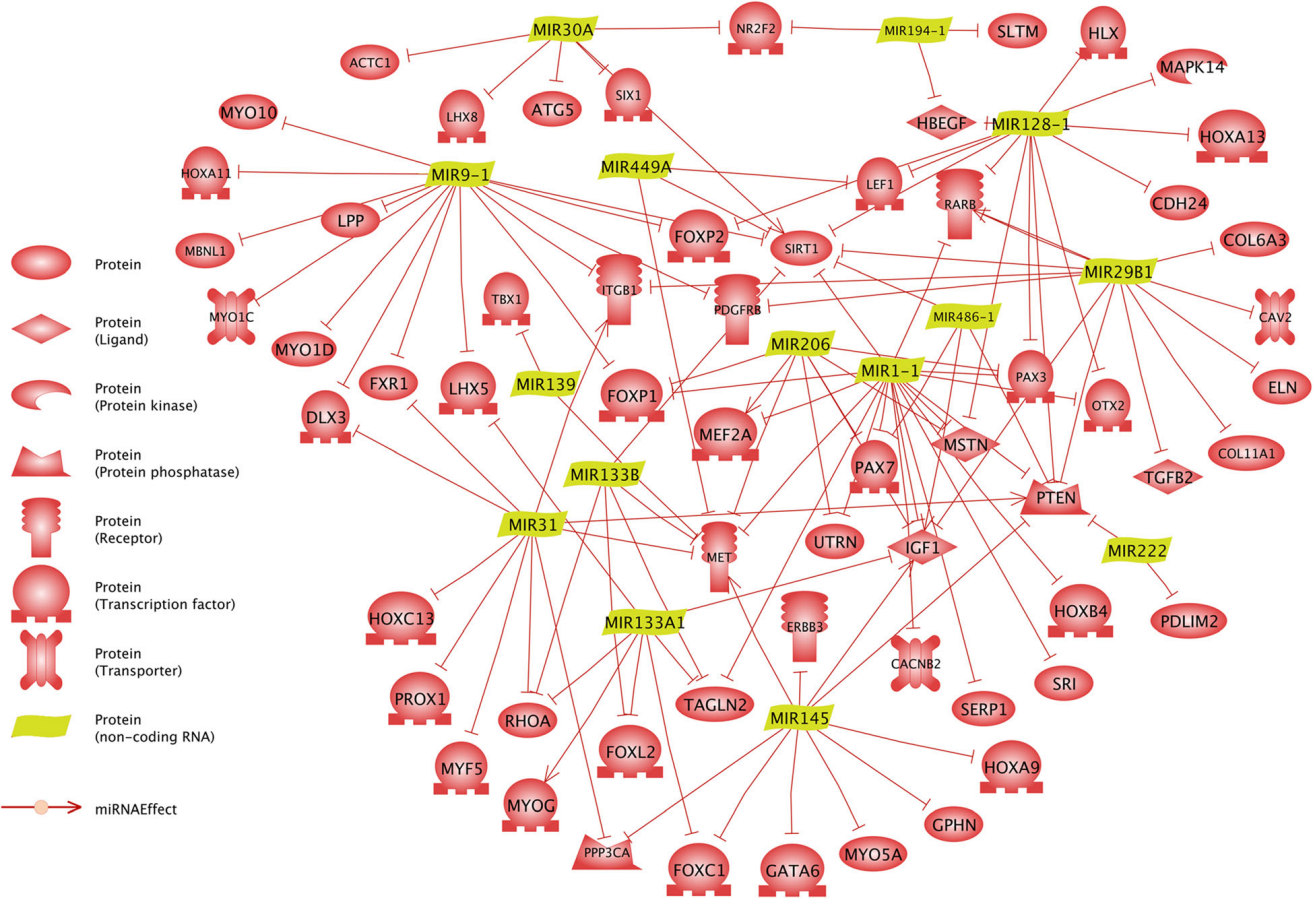
Relevance network of identified miRNAs and target genes belonging to the muscle organ development process (Pathway Studio Web, Elsevier, Netherlands)

A functional analysis of 1249 unique target genes predicted for 23 HER/LIM-specific miRNAs has shown nearly 200 processes in which they could be involved (DAVID, FDR ≤ 0.05). Among them is the muscle organ development process covering 40 target genes. Additionally, 21 genes were indicated in the PANTHER database to be participants of the same muscle organ development process (Table 3). Surprisingly, *Mef2a*, *Pax3*, *Pax7*, *Mbnl1*, and *Cacnb2* were the only target genes common for both analyses. Based on aforementioned findings, a relevance network of the direct interactions of miRNAs and their targets was prepared in the Pathway Studio Web software (Elsevier, Netherlands). It shows a complex network of the miRNA regulation of their direct or indirect target genes, some of which are well-known as myogenesis process-associated genes (Fig. 7). We suppose that the modulation of the expression of these genes (56 in total) by the HER/LIM cell-specific miRNA could govern the differences in myotube formation seen in the HER/LIM primary cultures. However, because this is only a prediction, their effective involvement in this phenomenon needs to be checked.

In addition to the aforementioned muscle organ development process, general processes such as positive and negative regulation of differentiation were also identified (Additional file 5). This indicates a putative capability of the identified miRNAs in the regulation of dozens of genes undergoing expression at this stage of in vitro myogenesis and underlines the complexity of miRNA-dependent post-translational modifications in the primary cultures of muscle cells.

## Conclusions

Thus far, this is the first study showing the interbreed comparison of miRNA expression in a primary culture of skeletal muscle cells subjected to myogenic differentiation; these cells were isolated from semitendinosus muscle of bulls of different breeds and performance. We demonstrated an increase in the number of myotubes on day 6 of the differentiation of muscle-derived cells of the HER/LIM beef breed origin as compared to that in the case of the HF dairy breed-related cells. Further, differences in the miRNA expression in these cultures were demonstrated.

Because the primary cultures were not pure and contained breed-dependent number of myogenic/non-myogenic cells, the net effect of the identified miRNA action should be considered to be the resultant effect of the activity and mutual interactions of satellite cells and muscle stem cell niche cells, which putatively led to the formation of a relatively large number of myotubes in beef cattle-related cells (HER/LIM) during in vitro myogenesis.

As the number of specific cells was not equalized in the primary cultures among breeds (a proportional increase in the number of satellite cell numbers brought about by four iterations of preplating), it is possible that the variation in the number of myotubes and the identified miRNA expression differences between HER/LIM and HF were the effect of the breed-specific cell content in the primary cultures of the skeletal muscle-derived cells. A co-culture system in which myogenic cells and other residents of the stem cell niche will be cultured separately but with the possibility to interact with each other would be a great opportunity to validate the obtained results. It would allow the identification of the myogenic or non-myogenic sources of the identified miRNA, by checking the examined cells separately. It would also be interesting to confirm the aforementioned interbreed miRNA expression differences in an in vivo study of the different stages of bovine myogenesis.

## Additional files

Additional file 1: Table S1. Real-time qPCR primers for selected genes. (XLS 38 kb)

Additional file 2: Table S2. Differences in miRNA expression in primary cultures of skeletal muscle cells originating from both HER/LIM breeds, when compared to the HF breed (FDR ≤ 0.05). (XLS 69 kb)

Additional file 3: Table S3. Spearman’s correlation coefficients (A) and their statistical importance (B) for qPCR-validated miRNAs (GenEx 6.0; MultiD Analyses AB, Sweden). (PDF 234 kb)

Additional file 4: Table S4. Target gene prediction for identified miRNAs (TargetScan; Pathway Studio Web, Elsevier, Netherlands). (XLS 2245 kb)

Additional file 5: Table S5. Functional analysis of miRNA target genes using the DAVID database (BP - biological processes). (XLS 145 kb)

Additional file 6: Table S6. miRNA target genes belonging to the muscle organ development process (PANTHER). (XLS 43 kb)

Additional file 7: Table S7. Functional analysis of miRNA target genes using KEGG (DAVID). (XLS 58 kb)

Additional file 8: Figures S1–S6. Selected pathways in which the identified target genes are involved (KEGG). (PDF 819 kb)

## Abbreviations

DAVID: The Database for Annotation, Visualization and Integrated Discovery
FC: Fold Change
FDR: False Discovery Rate
HER: Hereford
HF: Holstein-Friesian
KEGG: Kyoto Encyclopedia of Genes and Genomes
LIM: Limousin
NCBI GEO: National Center for Biotechnology Information Gene Expression Omnibus database
PANTHER: Protein ANalysis THrough Evolutionary Relationships Classification System
RIN: RNA Integrity Number
SEM: Standard Error of the Mean
