# Supplementary material for: Breed-dependent microRNA expression in the primary culture of skeletal muscle cells subjected to myogenic differentiation
Source: BMC Genomics. 2018 Jan 31;19:109. doi: 10.1186/s12864-018-4492-5 (PMC5793348; doi:10.1186/s12864-018-4492-5)
Supplement: Supplementary file 3 — Spearman’s correlation coefficients (A) and their statistical importance (B) for qPCR-validated miRNAs (GenEx 6.0; MultiD Analyses AB, Sweden). (PDF 234 kb) [file 12864_2018_4492_MOESM3_ESM.pdf]

Additional file 3: Table S3. Spearman's correlations coefficients (A) and their statistical importance (B) for qPCR validated miRNAs (GenEx 6.0; MultiD Analyses AB, Sweden).

A)	MYOGENIN	miR-1	miR-133a	miR-145	miR-206	miR-486	miR-139-5p	miR-9-5p	miR-503	miR-128	miR-660
MYOGENIN	1,00	0,67	0,69	-0,65	0,63	0,70	0,52	-0,59	0,38	0,54	0,26
miR-1	0,67	1,00	0,97	-0,69	0,95	0,80	0,84	-0,56	0,67	0,88	0,62
miR-133a	0,69	0,97	1,00	-0,63	0,97	0,82	0,85	-0,56	0,66	0,91	0,63
miR-145	-0,65	-0,69	-0,63	1,00	-0,55	-0,65	-0,45	0,68	-0,22	-0,60	-0,40
miR-206	0,63	0,95	0,97	-0,55	1,00	0,81	0,89	-0,46	0,73	0,89	0,64
miR-486	0,70	0,80	0,82	-0,65	0,81	1,00	0,83	-0,49	0,58	0,82	0,66
miR-139-5p	0,52	0,84	0,85	-0,45	0,89	0,83	1,00	-0,39	0,81	0,82	0,60
miR-9-5p	-0,59	-0,56	-0,56	0,68	-0,46	-0,49	-0,39	1,00	0,00	-0,50	-0,15
miR-503	0,38	0,67	0,66	-0,22	0,73	0,58	0,81	0,00	1,00	0,62	0,50
miR-128	0,54	0,88	0,91	-0,60	0,89	0,82	0,82	-0,50	0,62	1,00	0,79
miR-660	0,26	0,62	0,63	-0,40	0,64	0,66	0,60	-0,15	0,50	0,79	1,00

B)	MYOGENIN	miR-1	miR-133a	miR-145	miR-206	miR-486	miR-139-5p	miR-9-5p	miR-503	miR-128	miR-660
MYOGENIN	0,00E+00	3,39E-04	1,66E-04	5,58E-04	9,16E-04	1,60E-04	9,87E-03	2,31E-03	6,56E-02	6,09E-03	2,27E-01
miR-1	3,39E-04	0,00E+00	2,15E-14	2,03E-04	5,28E-13	2,51E-06	2,31E-07	4,13E-03	3,88E-04	1,70E-08	1,33E-03
miR-133a	1,66E-04	2,15E-14	0,00E+00	9,79E-04	1,51E-14	7,33E-07	2,00E-07	4,43E-03	4,29E-04	7,13E-10	1,00E-03
miR-145	5,58E-04	2,03E-04	9,79E-04	0,00E+00	5,86E-03	6,20E-04	2,55E-02	2,26E-04	2,92E-01	2,16E-03	5,22E-02
miR-206	9,16E-04	5,28E-13	1,51E-14	5,86E-03	0,00E+00	1,25E-06	7,66E-09	2,34E-02	5,90E-05	5,89E-09	6,78E-04
miR-486	1,60E-04	2,51E-06	7,33E-07	6,20E-04	1,25E-06	0,00E+00	4,10E-07	1,42E-02	3,13E-03	8,97E-07	4,09E-04
miR-139-5p	9,87E-03	2,31E-07	2,00E-07	2,55E-02	7,66E-09	4,10E-07	0,00E+00	5,77E-02	1,68E-06	1,19E-06	1,92E-03
miR-9-5p	2,31E-03	4,13E-03	4,43E-03	2,26E-04	2,34E-02	1,42E-02	5,77E-02	0,00E+00	9,97E-01	1,36E-02	4,75E-01
miR-503	6,56E-02	3,88E-04	4,29E-04	2,92E-01	5,90E-05	3,13E-03	1,68E-06	9,97E-01	0,00E+00	1,24E-03	1,21E-02
miR-128	6,09E-03	1,70E-08	7,13E-10	2,16E-03	5,89E-09	8,97E-07	1,19E-06	1,36E-02	1,24E-03	0,00E+00	4,61E-06
miR-660	2,27E-01	1,33E-03	1,00E-03	5,22E-02	6,78E-04	4,09E-04	1,92E-03	4,75E-01	1,21E-02	4,61E-06	0,00E+00
