# Supplementary figures and images for: Breed-dependent microRNA expression in the primary culture of skeletal muscle cells subjected to myogenic differentiation
Source: BMC Genomics. 2018 Jan 31;19:109. doi: 10.1186/s12864-018-4492-5 (PMC5793348; doi:10.1186/s12864-018-4492-5)

Figure S1

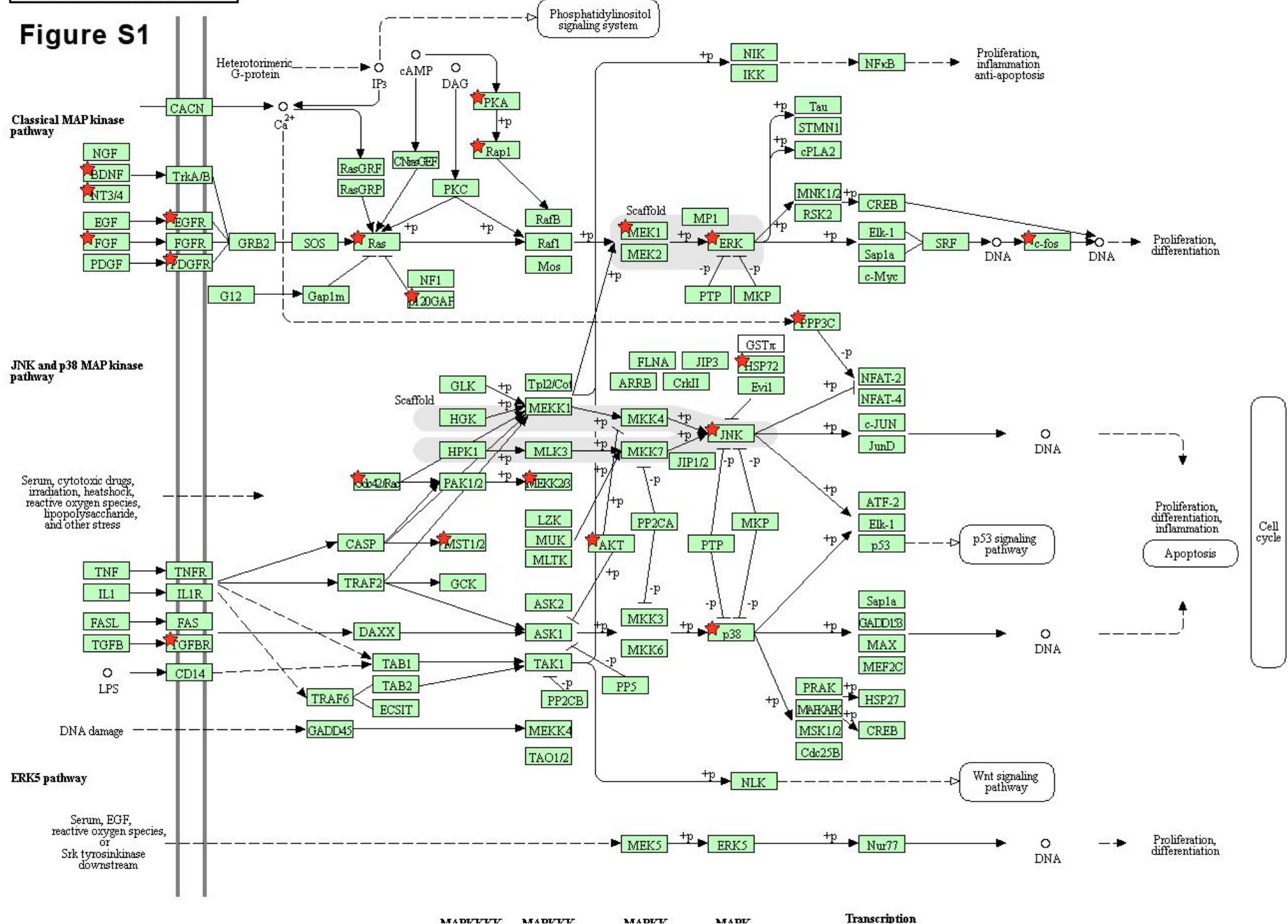

Figure S2

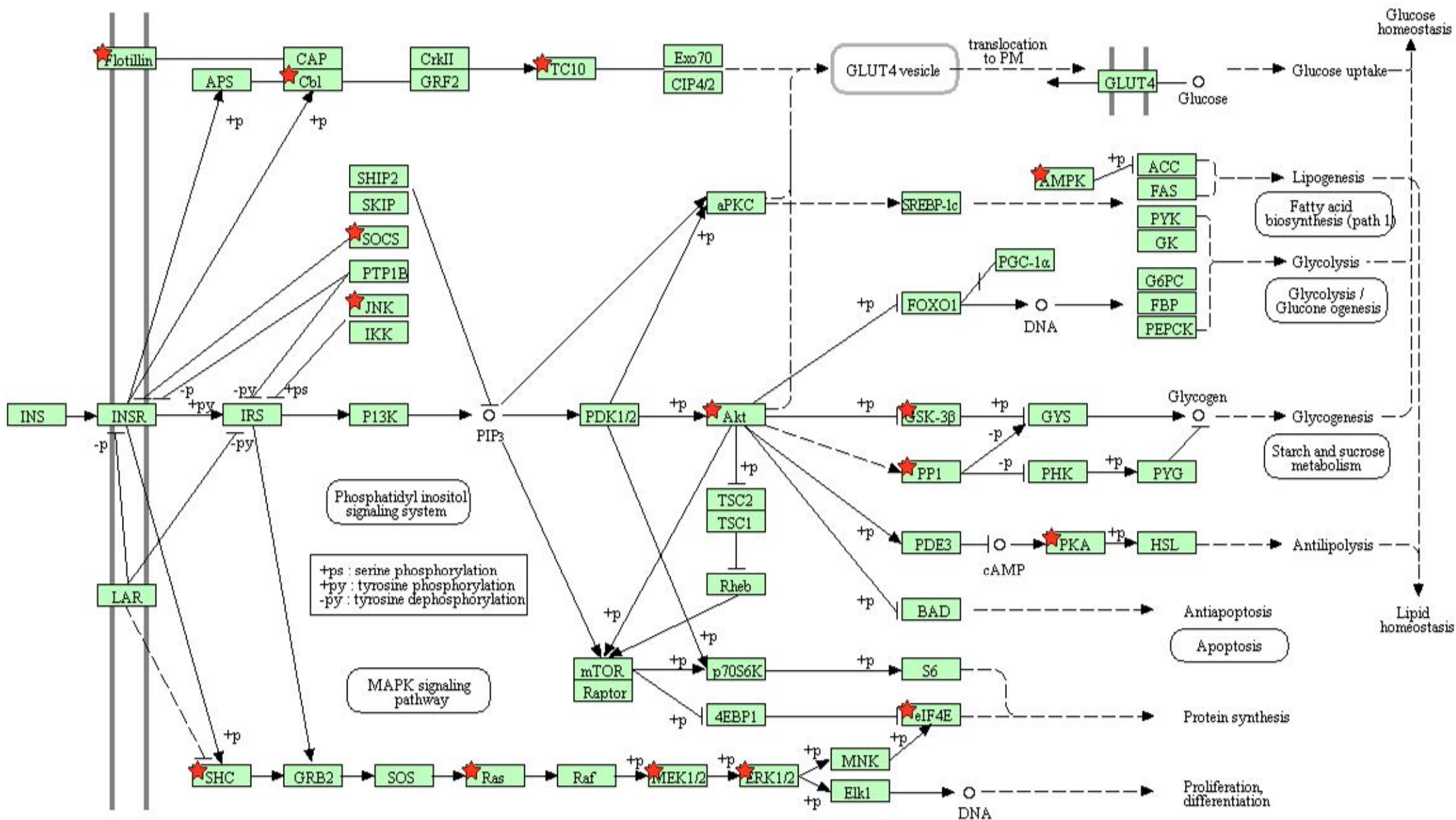

# mTOR SIGNALING PATHWAY

Figure S3

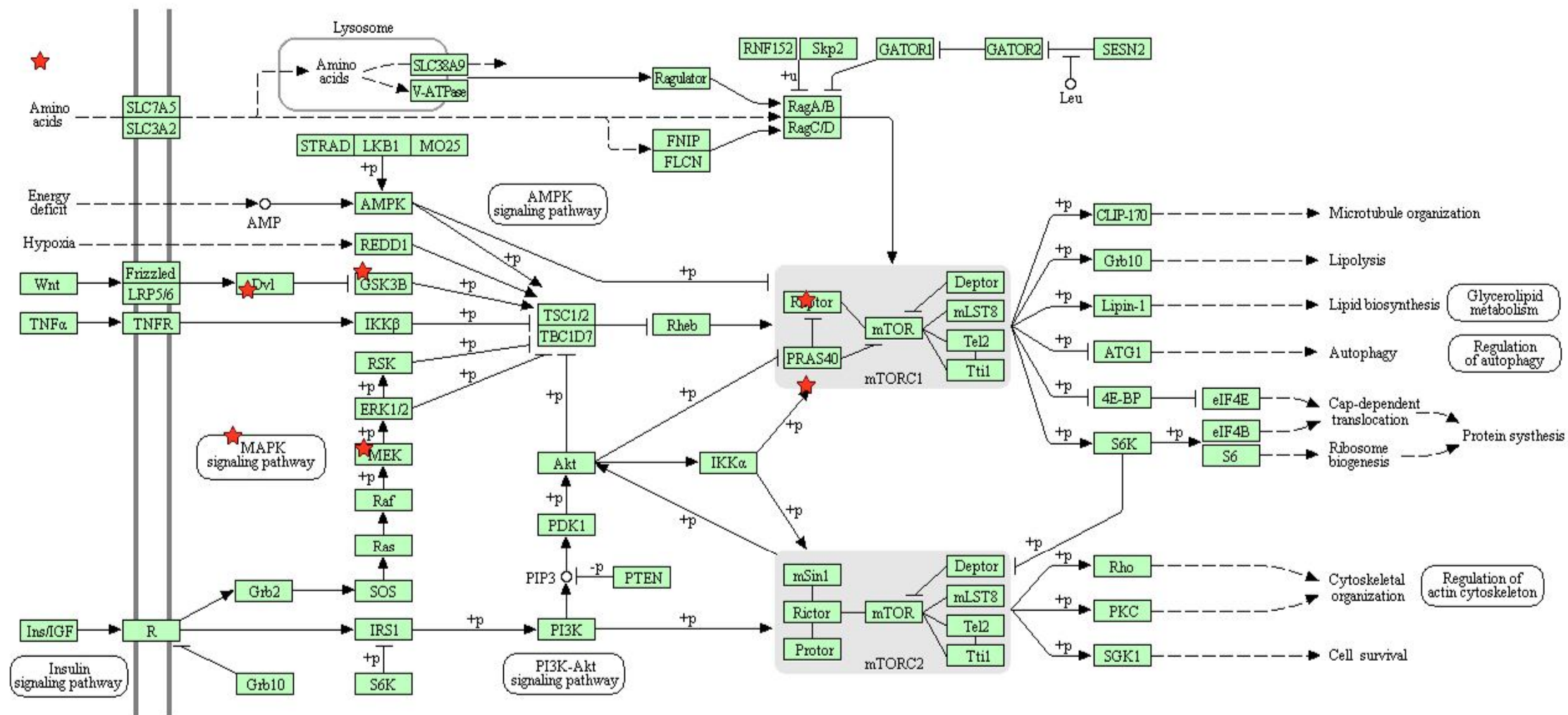

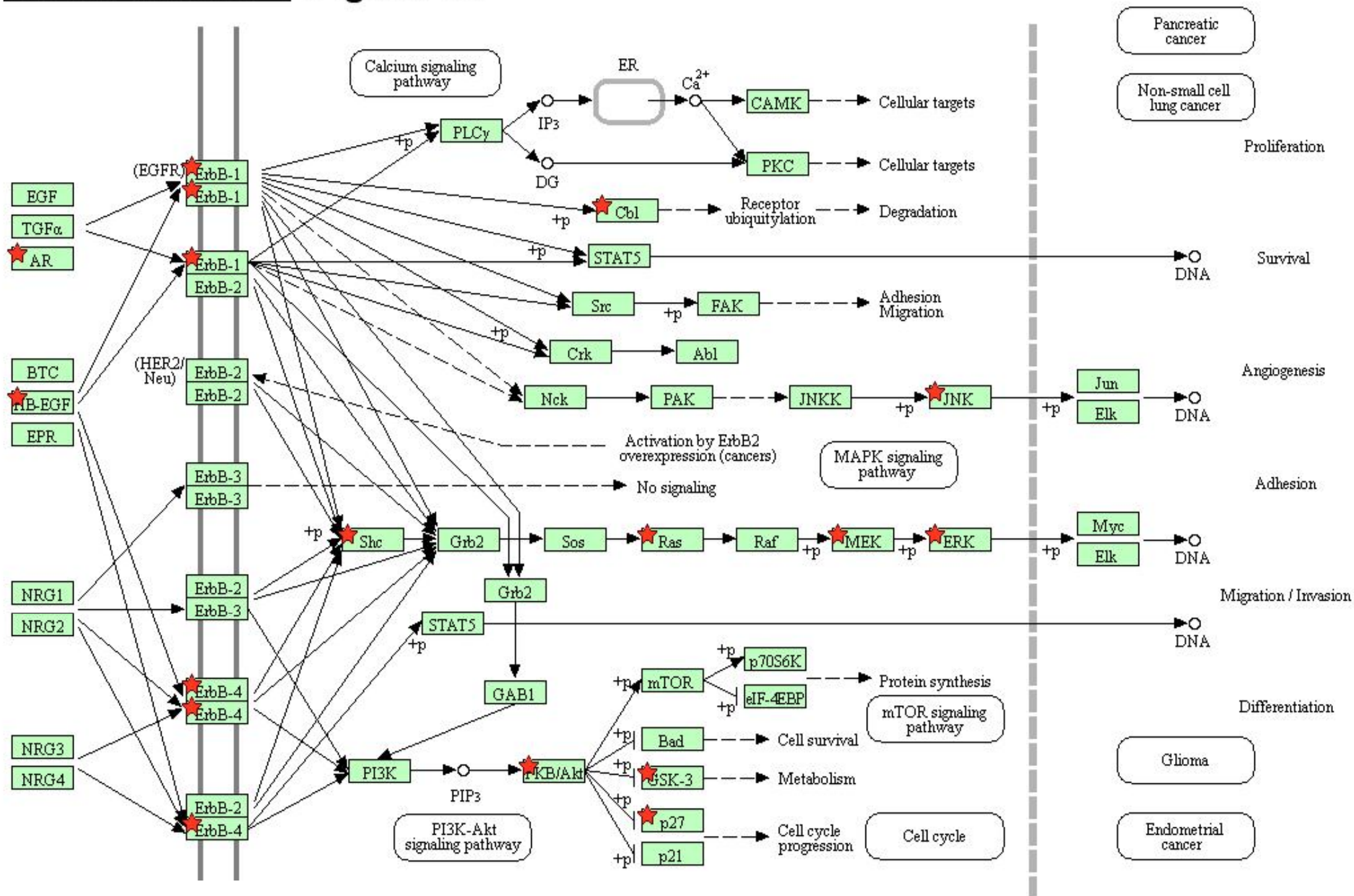

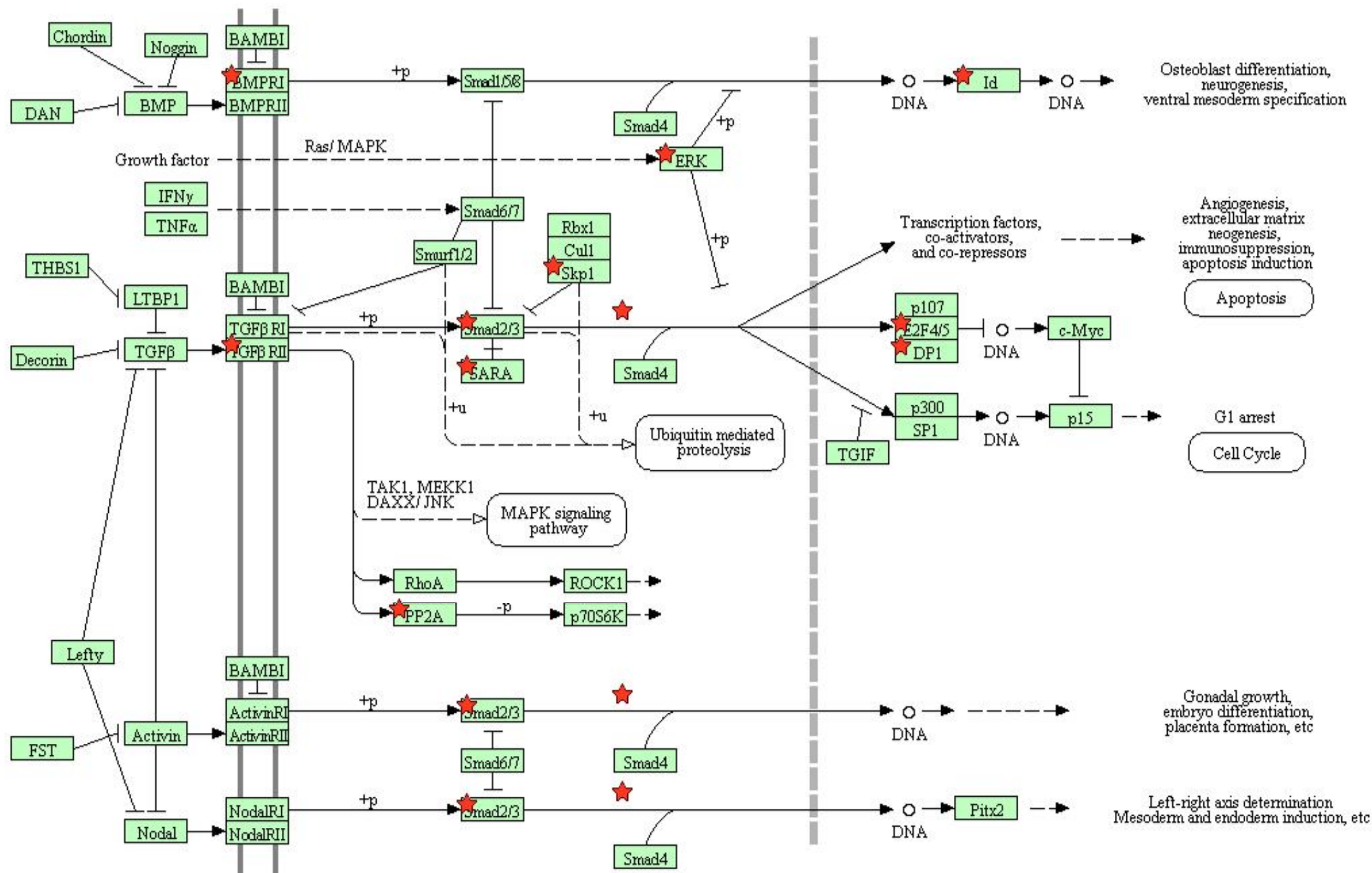

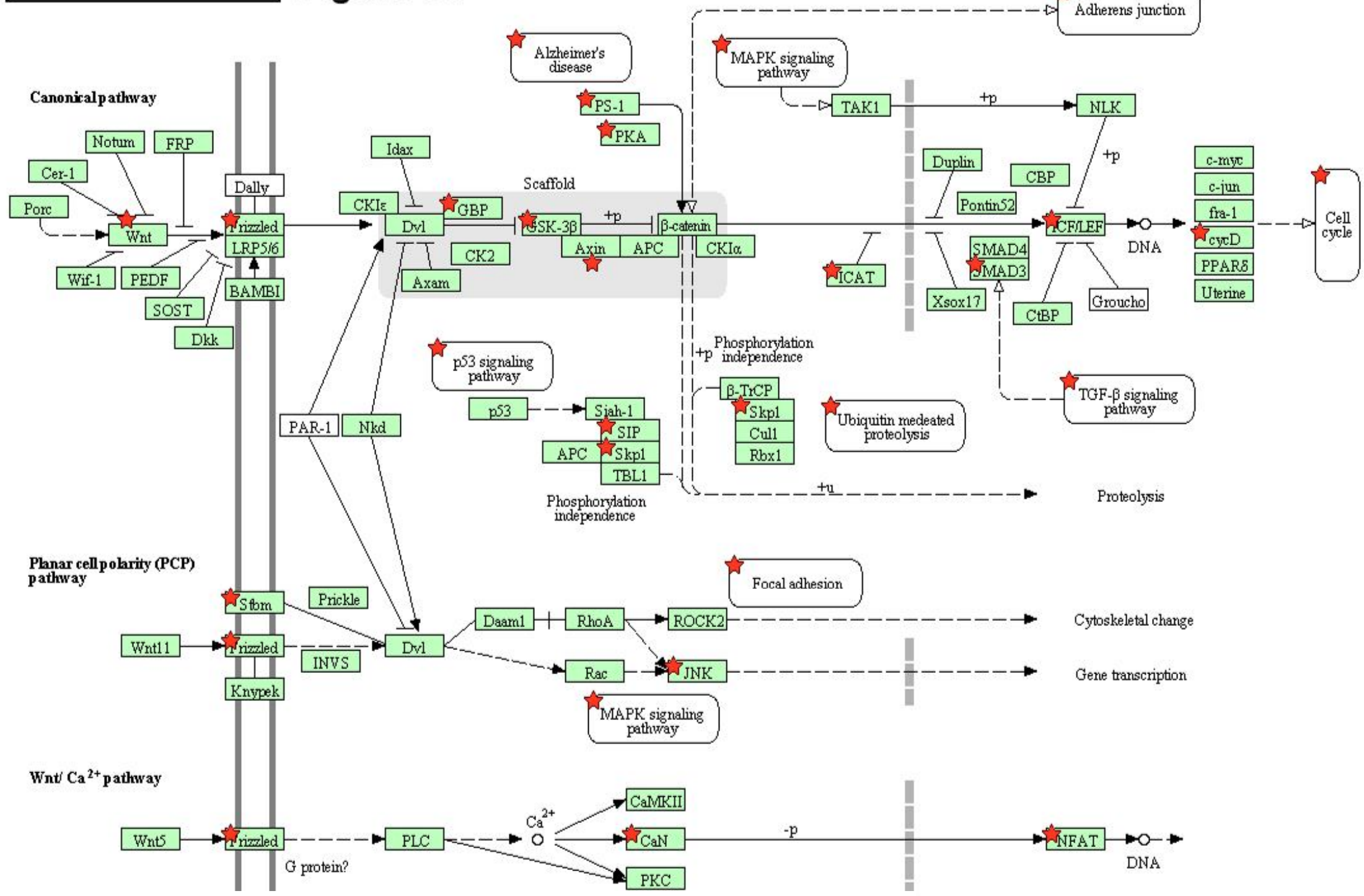

Supplement: Supplementary file 8 — Selected pathways in which the identified target genes are involved (KEGG). (PDF 819 kb) [file 12864_2018_4492_MOESM8_ESM.pdf]
